# Risk factors for non-vertebral fractures in community-dwelling elderly: a 10-year follow-up study in New Zealand

**DOI:** 10.1007/s11657-025-01530-7

**Published:** 2025-04-09

**Authors:** Haixia Liu, Zhenqiang Wu, Robert Scragg

**Affiliations:** 1 Section of Epidemiology & Biostatistics, School of Population Health, Faculty of Medical and Health Science, University of Auckland, Auckland, New Zealand; 2 Department of Epidemiology and Health Statistics, School of Public Health, Anhui Medical University, Hefei, China; 3 Department of Public Health and General Medicine, School of Life Sciences, Anhui University of Chinese Medicine, Hefei, China

**Keywords:** Non-vertebral fractures, Fracture prevention, Community-dwelling elderly, Cohort study, Risk factor

## Abstract

***Summary*:**

This 10-year study of 5000 + adults aged 50–84 found 20% experienced non-vertebral fractures. Higher risk was linked to female sex, older age, European ethnicity, lower education, living alone, alcohol use, prior falls/fractures, osteoporosis, arthritis, and antidepressants. Targeting modifiable factors (living arrangements, alcohol, antidepressants) could reduce fracture burden cost-effectively in older adults.

**Background:**

Although there has been extensive research on non-vertebral fractures, their risk factors remain incompletely understood. This study aimed to examine risk factors associated with non-vertebral fractures through a longitudinal examination of a community-dwelling cohort.

**Methods:**

This was a follow-up of participants recruited from family practices into a randomized trial of vitamin D supplementation and interviewed between 2011 and 2012, with follow-up until 2022. The outcome was the first non-vertebral fracture during the follow-up period, as identified from hospital events and insurance claims for fractures. Candidate risk factors were selected using a domain-based approach, and Cox models were employed to estimate adjusted hazard ratios (HRs).

**Results:**

The analysis comprised 5108 participants aged 50–84 years. Of these, 83% were of European/other ethnicity. A substantial proportion reported living with non-family members or living alone (20.5%), engaging in daily drinking (21.6%), or using antidepressants (11.9%). Over a median 10-year follow-up, 1016 participants (20%) experienced non-vertebral fractures. In the multivariable model, several factors were related to higher risk of non-vertebral fracture, including females (HR = 1.53), aged 80–84 years (HR = 1.47), European/other ethnicity, primary school education (HR = 1.65), living with non-family members (HR = 1.47) or living alone (HR = 1.29), daily alcohol drinking (HR = 1.51), history of falls (HR = 1.59) or fractures (HR = 1.43), osteoporosis (HR = 1.95), and arthritis (HR = 1.20), and dispensing of antidepressants (HR = 1.52) and antiarrhythmic medications (HR = 1.51).

**Conclusion:**

Non-vertebral fractures are prevalent among older adults, with several prevalent and potentially modifiable risk factors identified, such as living situation, drinking habits, and antidepressant dispensing. Further exploration of these factors’ causality and the implementation of public health interventions targeting them, could yield significant benefits and cost-effectively reduce the burden of fractures.

**Trial registration:**

This study was registered with the Australian New Zealand Clinical Trials Registry (ACTRN12611000402943).

**Supplementary Information:**

The online version contains supplementary material available at 10.1007/s11657-025-01530-7.

## Introduction

With a rising life expectancy and an increasing number of elderly adults, fractures have become a significant public health problem globally, contributing to high morbidity rates, mortality rates, and socioeconomic burden [1–5]. The Global Burden of Disease Study (GBD) 2019 compared global fracture incidence, prevalence, and years lived with disability (YLDs) across the 21 GBD regions and 204 countries and territories, where there were 178 million new fractures, 455 million prevalent cases of acute or long-term fracture symptoms, and 25.8 million YLDs in 2019, an increase of 33.4%, 70.1%, and 65.3% since 1990, respectively [1]. In the US population ≥ 50 years of age, more than 2 million incident fractures were estimated to have occurred in 2005 (73% were non-vertebral fractures), costing $17 billion (94% from non-vertebral fractures). Projections suggest that by 2025, annual fractures and associated costs will increase by almost 50%, with over 3 million fractures incurring $25.3 billion in costs [2]. In the largest five countries of the European Union plus Sweden, total fragility fractures (non-vertebral fractures accounted for 84.5%) were estimated to increase 23%, from 2.7 million in 2017 to 3.3 million in 2030, and the resulting annual fracture-related costs were expected to increase 27%, from €37.5 billion in 2017 to €47.4 billion in 2030 [3]. All major types of fragility fractures and even minor fractures with older age were observed to be associated with increased mortality, which persisted after the fracture for 5 years for all fractures and up to 10 years for hip fractures [4, 5].

Many studies consistently indicate an increase in fracture incidence with age for both sexes, especially in older adults aged ≥ 80 years and females [6–9]. Similarly, a substantial body of evidence suggests that the risk of fracture varies considerably according to regional ethnicity, personal and family circumstances, physical health, medical history, drugs, and behaviors [8–21]. However, this evidence is primarily derived from studies of vertebral fractures or hip fractures [8, 10–13, 15–18, 20, 21], despite non-hip non-vertebral fractures accounting for most fractures, affecting a large proportion of the population and being linked with adverse outcomes [22–24]. For non-vertebral fracture studies, bone mineral density (BMD) and pharmacological treatment usually have been the main focus [25–27]; only a few studies explored other influencing factors [8, 9, 15], and most of them had a relatively short follow-up time, fewer available baseline factors, and small sample size, which warrants further research.

Given the significant burden of non-vertebral fractures and the constraints of long-term follow-up cohort studies, this study investigated risk factors of non-vertebral fractures by utilizing the extended follow-up of an older adult cohort (from 2011 to 2022) from the Vitamin D Assessment (ViDA) study [28].

## Methods

### Study design and population

This study was an extended 10-year prospective follow-up (2011–2022) of the ViDA study participants, who were recruited from community family practices from April 2011 to November 2012 in Auckland, New Zealand. The ViDA study was a randomized, double-blind, placebo-controlled trial to evaluate the efficacy of oral monthly 100,000 IU vitamin D_3_ supplementation or identical placebo (after 200,000 IU of vitamin D bolus initially) on cardiovascular disease (CVD) from 2011 to 2015. The inclusion criteria were the following: (1) age 50–84 years, (2) ability to give informed consent, (3) resident in Auckland at recruitment and anticipated residence in New Zealand for the study period. The exclusion criteria were the following: (1) current use of vitamin D supplements (> 600 IU per day if aged 50–70 years, > 800 IU per day if aged 71–84 years); (2) diagnosis of psychiatric disorders in the last 2 years that would limit ability to comply with study protocol; (3) a history of hypercalcemia, nephrolithiasis, sarcoidosis, parathyroid disease, or gastric bypass surgery; (4) enrolled in another study which could affect participation in the vitamin D study; (5) baseline serum calcium concentration > 2.5 mmol/L. During the active intervention stage of the ViDA trial, monthly 100,000 IU vitamin D supplementation did not prevent falls (HR = 0.99, 95% CI = 0.92–1.07) or non-vertebral fractures (HR = 1.19, 95% CI = 0.94–1.50) over a median of 3.3 years intervention period. Given the short half-life of native vitamin D in blood circulation, it is unlikely that there would be any lasting effects from the supplementation. Therefore, participants from both intervention and placebo groups were combined for the purpose of the current risk factor analysis. Details of the study methods and main trial results were published elsewhere [28, 29]. The ethics approval was given by the New Zealand Multi-region Ethics Committee (MEC/09/08/082), and all participants gave written informed consent. The study was registered with the Australian New Zealand Clinical Trials Registry (ACTRN12611000402943).

### Baseline interview

The baseline characteristics include the following: (1) sociodemographic variables (sex; age; ethnic group: European/other, Māori, Pacific, South Asian; highest education level: primary, secondary, tertiary (e.g., university); current employment: paid, retired, other; current marital status: married/partnered, separated/divorced/living alone, widow/widower, never married/partnered; type of accommodation: house/flat/apartment, retirement village, other; living situation: family members, non-family members, alone), (2) lifestyle (tobacco smoking: current smoker, ex-smoker, and never smoker; alcohol drinking frequency in the last 12 months: never drinking, less than four times monthly, less than seven times weekly, and daily drinking; vigorous physical activity (hours per week): none, 1–2, > 2), (3) physical health (self-reported health status: excellent/very good, good, fair/poor), and (4) self-reported medical history that is diagnosed by a doctor (bone health: fall in the last 4 weeks, previous fracture, osteoporosis, and arthritis; CVD: heart attack and angina, heart failure and irregular heart beat (IHB), stroke and transient ischemic attack (TIA), diabetes, high blood pressure, high cholesterol level; and other diseases: asthma, emphysema, psoriasis, eczema, cancer, chronic pain, and depression).

The New Zealand index of socioeconomic deprivation (NZDep), which measures the level of deprivation in a small area (ranging from 1 to 10) [30], was captured by linking each participant’s unique identifier—National Health Index (NHI)—with the Ministry of Health of New Zealand (MoH) National Health Index database. Weight (nearest 0.1 kg) and height (nearest 0.1 cm) were measured without shoes and in light clothing by trained staff. Body mass index (BMI) was calculated by the weight (in kilograms) divided by height (in meters) squared. Participants were classified into underweight (BMI < 18.5 kg/m^2^), normal weight (BMI = 18.5–24.9 kg/m^2^), overweight (BMI = 25.0–29.9 kg/m^2^), and obese (BMI ≥ 30.0 kg/m^2^) in the analysis. A 25 mL non-fasting blood sample was collected for initial measurement of serum calcium to screen for hypercalcemia, and then for later measurement of 25(OH)D_3_ and 25(OH)D_2_ concentrations in serum stored at − 80 °C. Deseasonalized 25(OH)D concentrations were estimated by eliminating a sinusoidal component from baseline 25(OH)D values of all participants [31].

### Prescription medications

The prescription medications of each participant were extracted from the MoH pharmaceutical database, which contains the chemical name, quantity dispensed, daily dosage, and frequency. Based on the Anatomical Therapeutic Chemical (ATC) level 2 category, the baseline prescription of medications (defined as within 12 months prior randomization) was categorized into the following categories (the specific chemical name of medications included in the analyses are shown in Suppl Table 1):

- Musculoskeletal medications (non-steroidal anti-inflammatory drugs)
- Nervous system medications (antiepileptic medication, analgesic drugs (non-opioid or opioid analgesics), antidepressants, antipsychotics, anxiolytics, and sedatives and hypnotic drugs)
- CVD medications (agents affecting the renin-angiotensin system, alpha-adrenoceptor blockers, antiarrhythmic medications, beta-adrenoceptor blockers, calcium channel blockers, diuretics, nitrates, and lipid-lowering agents)
- Other medications including respiratory medications (antihistamines and anticholinergic agents) and alimentary tract medications (laxatives)

### Outcome and follow-up

The outcome of interest in this analysis was the first non-vertebral fracture for each participant during the follow-up period, which was identified from two sources: the MoH National Minimum Dataset (NMDS) of hospital events for fractures and the Accident Compensation Corporation (ACC) data of claims for fractures.

The NMDS is a national collection of public and private hospital discharge information which was used to track hospital discharges. The non-vertebral fractures from NMDS were defined as hospital discharges with primary or secondary diagnoses of non-vertebral fractures relevant to ICD-10 codes (see details in Suppl Table 2). The ACC is the national governmental insurance organization that covers all New Zealand residents and visitors for any medical and hospital costs from injury. The non-vertebral fractures from the ACC were defined as claims made after randomization with descriptions of non-vertebral fractures, and relevant ICD-10 codes or Read codes (Suppl Table 2).

The deaths of participants were identified using the MoH Mortality Collection. The follow-up period was defined as from each participant’s date of randomization to 31 August 2022.

### Statistical analysis

Several Cox proportional hazards regression models, with estimated hazard ratios (HR) and 95% confidence intervals (95% CI), were used to explore the relationship between baseline risk factors and non-vertebral fracture. Baseline factors of interest were broadly divided into sociodemographic, lifestyle, and physical health and physical/laboratory measurements, medical history (bone health, CVD, and other diseases), and prescription medications (musculoskeletal medications, nervous system medications, CVD medications, and other medications). Follow-up years from randomization to the earliest of either the end of follow-up (31 August 2022), the first non-vertebral fracture event, or death were used as the time scale for all time-to-event analyses. A step-by-step domain-based method was used to guide the selection of potential risk factors:

- Step 1: Pre-selected variables of interest were categorized into specific domains, including sociodemographic, lifestyle, physical health and physical/laboratory measurements, medical history, and medications.
- Step 2: A multivariable model was constructed by incorporating all significant variables from the sociodemographic domain identified in the univariate analysis (at a pre-selected 0.10 significant level); non-significant variables were then excluded to form the base model (Model 1).
- Step 3: Within each remaining domain, variables were individually added to Model 1, resulting in a series of models for each domain (Model 2); all significant variables from Model 2 (at a pre-selected 0.10 significant level) were added to Model 1, forming a comprehensive model for each domain (Model 3).
- Step 4: All significant variables (at a pre-selected 0.10 significant level) from each domain in Model 3 were used to build the final model (Model 4).

Rare medical history (e.g., carotid artery stenosis) and medications (e.g., anti-rheumatoid agents, muscle relaxants, and anti-Parkinson agents), which were defined < 1% in our studied sample, were excluded from the analysis. Instead of including all significant factors from Model 3, a sensitivity analysis with stepwise selection was conducted for the final model. The multicollinearity of all factors from Model 3 was also examined, and the results indicated low multicollinearity concern (all tolerance values > 0.10). All analyses were performed using SPSS version 23.0 (IBM Co., Armonk, NY, USA), and *p*-value < 0.05 (two-tailed test) was considered statistically significant in the final model.

## Results

**Fig. 1:**
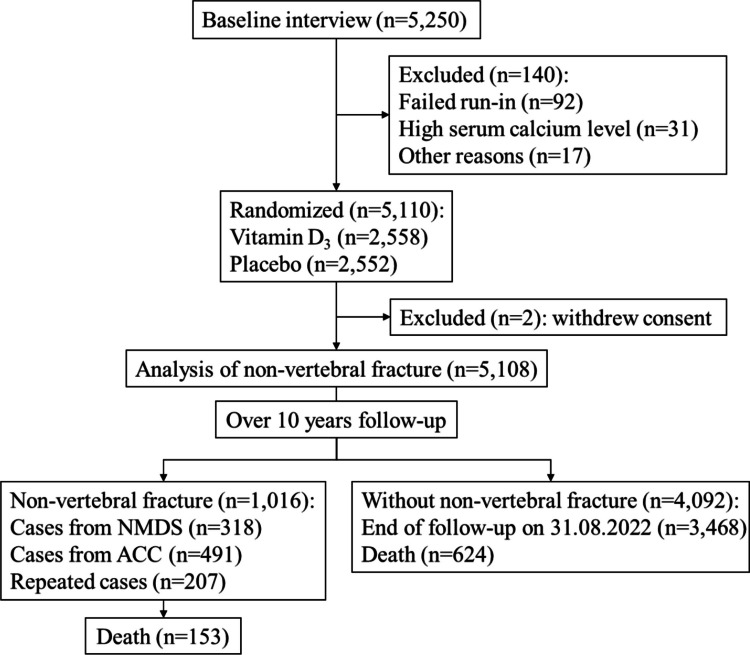
The flow diagram of eligible participants and identification of outcomes in this study

A total of 5110 participants were randomized to either monthly 100,000 IU vitamin D supplementation (*n* = 2558) or identical placebo (*n* = 2552) for a median of 3.3 years from 2011 to 2015. Two participants initially assigned to the placebo group withdrew consent after randomization and were excluded from the analysis. The 5108 participants left were included in the analysis of fractures, with an extended follow-up until 31 August 2022 (Fig. 1). Over the median 10 years (IQR = 8.3–10.7) of follow-up, 1016 were identified as having non-vertebral fractures (19.9%, 318 from MoH NMDS, 491 from ACC claims, and 207 for both), and 777 deaths (15.2%) occurred including 153 deaths in participants after an initial fracture (Fig. 1).

Among the 5108 participants, there were more males (58.1%) than females (41.9%). The mean (SD) age of participants was 65.9 (8.3) years. Most of the participants identified as European or other (83.3%), followed by Pacific (6.5%), Māori (5.3%), and South Asian (4.9%). More than half of the participants had a tertiary education (56.4%) and were employed in paid positions (51.3%). Most participants were married or partnered (72.7%) and living with their family members (79.4%). The sociodemographic characteristics of the baseline are shown in Table 1.

**Table 1:** Sociodemographic characteristics of baseline and hazard ratio for non-vertebral fractures during follow-up

Sociodemographic characteristic: *n* (column%)	Non-vertebral fractures, *n* (row%)	Univariate Cox model	
		HR (95% CI), *p* value	*p* value*
Sex			< 0.001
Female: 2139 (41.9)	518 (24.2)	1.46 (1.29, 1.65), < 0.001	
Male: 2969 (58.1)	498 (16.8)	1.00	
Age (years)			< 0.001
50–59: 1138 (22.3)	200 (17.6)	1.00	
60–69: 2220 (43.5)	422 (19.0)	1.13 (0.96, 1.34), 0.16	
70–79: 1438 (28.2)	298 (20.7)	1.32 (1.10, 1.58), 0.002	
80–84: 312 (6.1)	96 (30.8)	2.30 (1.80, 2.93), < 0.001	
Ethnic group			< 0.001
European/other: 4253 (83.3)	929 (21.8)	1.00	
Māori: 272 (5.3)	38 (14.0)	0.64 (0.46, 0.88), 0.007	
Pacific: 334 (6.5)	29 (8.7)	0.37 (0.26, 0.54), < 0.001	
South Asian: 249 (4.9)	20 (8.0)	0.33 (0.21, 0.52), < 0.001	
Highest education level			0.07
Primary school: 95 (1.9)	25 (26.3)	1.50 (1.01, 2.25), 0.046	
Secondary school: 2127 (41.6)	437 (20.5)	1.10 (0.97, 1.24), 0.15	
Tertiary (eg. university): 2882 (56.4)	554 (19.2)	1.00	
Missing: 4 (0.1)	0		
Current employment			< 0.001
Paid: 2618 (51.3)	484 (18.5)	1.00	
Retired: 2059 (40.3)	450 (21.9)	1.30 (1.14, 1.48), < 0.001	
Other: 426 (8.3)	82 (19.2)	1.07 (0.85, 1.35), 0.57	
Missing: 5 (0.1)	0		
Current marital status			< 0.001
Married/partnered: 3714 (72.7)	671 (18.1)	1.00	
Separated/divorced/living alone: 645 (12.6)	149 (23.1)	1.33 (1.11, 1.59), 0.002	
Widow/widower: 440 (8.6)	122 (27.7)	1.73 (1.43, 2.10), < 0.001	
Never married/partnered: 304 (6.0)	74 (24.3)	1.43 (1.12, 1.81), 0.004	
Missing: 5 (0.1)	0		
Type of accommodation			0.009
House/flat/apartment: 4939 (96.7)	971 (19.7)	1.00	
Retirement village: 113 (2.2)	31 (27.4)	1.61 (1.13, 2.30), 0.009	
Other: 51 (1.0)	14 (27.5)	1.58 (0.93, 2.68), 0.09	
Missing: 5 (0.1)	0		
Living situation			< 0.001
Family members: 4057 (79.4)	729 (18.0)	1.00	
Non-family members: 107 (2.1)	30 (28.0)	1.69 (1.17, 2.43), 0.005	
Alone: 939 (18.4)	257 (27.4)	1.67 (1.45, 1.92), < 0.001	
Missing: 5 (0.1)	0		
NZDep			0.04
1–2: 1953 (38.2)	433 (22.2)	1.00	
3–4: 925 (18.1)	169 (18.3)	0.84 (0.71, 1.01), 0.06	
5–6: 870 (17.0)	165 (19.0)	0.86 (0.72, 1.03), 0.11	
7–8: 547 (10.7)	114 (20.8)	0.96 (0.78, 1.18), 0.69	
9–10: 804 (15.7)	135 (16.8)	0.76 (0.62, 0.92), 0.005	
Missing: 9 (0.2)	0		

*n* number of participants, *HR* hazard ratio, *95% CI* 95% confidence interval, *NZDep* the New Zealand index of socioeconomic deprivation

*p value from type 3 test

Only 6.3% were current smokers, and 42.6% were ex-smokers. In the last 12 months, more than 1 in 5 (21.6%) reported drinking alcohol daily. In a typical week during the past 3 months, 39.8% reported no vigorous physical activity. The majority of participants were overweight or obese (BMI ≥ 25.0 kg/m^2^), and most self-reported very good or excellent health status (75.5%). A total of 1755 (34.4%) participants reported having arthritis (34.4%) at baseline, 2378 (46.6%) had a previous fracture, 308 (6.0%) had a fall in the last 4 weeks, and 610 (11.9%) had been dispensed one or more antidepressants during 12 months prior to baseline interview (11.9%). Other baseline characteristics are shown in Suppl Tables 3–6.

A step-by-step risk factor examination was conducted to explore the potential risk factors for the non-vertebral fracture. A total of nine pre-selected sociodemographic factors were examined in the multivariable model, and five of them (sex, age, ethnic group, highest education level, and living situation) satisfied the pre-selected significance level and formed Model 1 (Table 2). Smoking status and alcohol frequency from the lifestyle domain remained significant in Model 3 (Suppl Table 3). Self-reported health status from physical health and physical/laboratory measurement domain remained significant (Suppl Table 4). Four factors (fall in the last 4 weeks, previous fracture, osteoporosis, arthritis) from bone health history and one factor (heart failure & irregular heartbeat) from CVD history remained significant in Model 3 (Suppl Table 5). In addition, a total of four prescription medication categories (antidepressants, alpha-adrenoceptor blockers, antiarrhythmic medications, and nitrates) remained significant in Model 3 and were identified as potential risk factors for non-vertebral fractures (Suppl Table 6).

**Table 2:** Sociodemographic characteristics and adjusted hazard ratios of non-vertebral fractures during follow-up

Sociodemographic characteristic: *n* (column%)	Multivariable Cox model (Model 1)	
	HR (95% CI), *p* value	*p* value*
Sex		< 0.001
Female: 2137 (41.9)	1.45 (1.28, 1.65), < 0.001	
Male: 2966 (58.1)	1.00	
Age (years)		< 0.001
50–59: 1135 (22.2)	1.00	
60–69: 2219 (43.5)	0.93 (0.78, 1.11), 0.41	
70–79: 1437 (28.2)	1.06 (0.88, 1.28), 0.52	
80–84: 312 (6.1)	1.73 (1.34, 2.24), < 0.001	
Ethnic group		< 0.001
European/Other: 4250 (83.3)	1.00	
Māori: 272 (5.3)	0.60 (0.43, 0.84), 0.003	
Pacific: 334 (6.5)	0.36 (0.25, 0.53), < 0.001	
South Asian: 247 (4.8)	0.38 (0.25, 0.60), < 0.001	
Highest education level		0.02
Primary school: 95 (1.9)	1.76 (1.17, 2.65), 0.007	
Secondary school: 2127 (41.7)	1.05 (0.93, 1.20), 0.42	
Tertiary (e.g., university): 2881 (56.5)	1.00	
Living situation		< 0.001
Family members: 4057 (79.5)	1.00	
Non-family members: 107 (2.1)	1.59 (1.10, 2.30), 0.01	
Alone: 939 (18.4)	1.39 (1.20, 1.61), < 0.001	

*HR* hazard ratio, adjusted for all other variables in the table; *95% CI* 95% confidence interval; the non-significant variables (at a preselected 0.10 significant level) were excluded from Model 1. A total of 5103 participants were included in the model (99.9% out of 5108)

*Adjusted *p* value from type 3 test

### Risk factors of non-vertebral fractures in the final model

As shown in Table 3, a total of 17 candidate risk factors from the four domains were examined in the final multivariable model (Model 4). Results showed a significantly higher risk of non-vertebral fracture with being female (HR = 1.53, 95% CI = 1.33–1.75), older age (80–84 years, HR = 1.47, 95% CI = 1.12–1.92), European/other ethnicity (Māori HR = 0.58, 95% CI = 0.41–0.83; Pacific HR = 0.44, 95% CI = 0.29–0.66; South Asian HR = 0.51, 95% CI = 0.32–0.81), primary school level education (HR = 1.65, 95% CI = 1.08–2.52), living with non-family member (HR = 1.47, 95% CI = 1.01–2.14) or living alone (HR = 1.29, 95% CI = 1.11–1.51), daily alcohol drinkers (HR = 1.51, 95% CI = 1.18–1.92), all four medical conditions related to bone health (fall in the last 4 weeks HR = 1.59, 95% CI = 1.29–1.97; previous fracture HR = 1.43, 95% CI = 1.26–1.63; osteoporosis HR = 1.95, 95% CI = 1.33–2.87; and arthritis HR = 1.20, 95% CI = 1.05–1.37), and prescription medications (antidepressants HR = 1.52, 95% CI = 1.28–1.79; antiarrhythmic medications HR = 1.51, 95% CI = 1.02–2.25). In addition, a linear trend was found between alcohol drinking frequency and the hazard of non-vertebral fractures (all *p* for trend < 0.001, Suppl Fig. 1). In the sensitivity analysis, we found similar results, the only change being for nitrates which became significant (HR = 1.35, 95% CI = 1.02–1.79, Suppl Table 7).

**Table 3:** Multivariable Cox model and adjusted hazard ratio of non-vertebral fractures during follow-up

Characteristic: *n* (column%)	Multivariable Cox model (final model)	
	HR (95% CI), *p* value	*p* value*
Sociodemographic		
Sex		< 0.001
Female: 2088 (41.9)	1.53 (1.33, 1.75), < 0.001	
Male: 2898 (58.1)	1.00	
Age (years)		< 0.001
50–59: 1123 (22.5)	1.00	
60–69: 2169 (43.5)	0.87 (0.73, 1.04), 0.12	
70–79: 1388 (27.8)	0.92 (0.76, 1.13), 0.44	
80–84: 306 (6.1)	1.47 (1.12, 1.92), 0.006	
Ethnic group		< 0.001
European/Other: 4154 (83.3)	1.00	
Māori: 260 (5.2)	0.58 (0.41, 0.83), 0.002	
Pacific: 328 (6.6)	0.44 (0.29, 0.66), < 0.001	
South Asian: 244 (4.9)	0.51 (0.32, 0.81), 0.004	
Highest education level		0.07
Primary school: 93 (1.9)	1.65 (1.08, 2.52), 0.02	
Secondary school: 2070 (41.5)	1.03 (0.90, 1.17), 0.70	
Tertiary (eg. university): 2823 (56.6)	1.00	
Living situation		0.001
Family members: 3976 (79.7)	1.00	
Non-family members: 104 (2.1)	1.47 (1.01, 2.14), 0.04	
Alone: 906 (18.2)	1.29 (1.11, 1.51), 0.001	
Lifestyle		
Tobacco smoking		0.12
Current smoker: 312 (6.3)	1.27 (0.98, 1.66), 0.07	
Ex-smoker: 2126 (42.6)	1.10 (0.96, 1.26), 0.15	
Never smoker: 2548 (51.1)	1.00	
Alcohol drinking frequency in the last 12 months		< 0.001
None: 694 (13.9)	1.00	
< 4 times monthly: 1510 (30.3)	1.06 (0.84, 1.34), 0.61	
< 7 times weekly: 1709 (34.3)	1.19 (0.94, 1.51), 0.15	
Daily: 1073 (21.5)	1.51 (1.18, 1.92), 0.001	
Physical health		
Self-reported health status		0.94
Excellent/very good: 3779 (75.8)	1.00	
Good: 1002 (20.1)	1.00 (0.85, 1.17), 0.96	
Fair/poor: 205 (4.1)	1.05 (0.77, 1.43), 0.75	
Medical history		
Bone health		
Fall in the last 4 weeks		< 0.001
Yes: 303 (6.1)	1.59 (1.29, 1.97), < 0.001	
No: 4683 (93.9)	1.00	
Previous fracture		< 0.001
Yes: 2322 (46.6)	1.43 (1.26, 1.63), < 0.001	
No: 2664 (53.4)	1.00	
Osteoporosis		0.001
Yes: 69 (1.4)	1.95 (1.33, 2.87), 0.001	
No: 4917 (98.6)	1.00	
Arthritis		0.007
Yes: 1723 (34.6)	1.20 (1.05, 1.37), 0.007	
No: 3263 (65.4)	1.00	
CVD		
Heart failure and IHB		0.25
Heart failure ± IHB: 83 (1.7)	1.32 (0.83, 2.11), 0.24	
IHB only: 604 (12.1)	1.14 (0.94, 1.39), 0.19	
Neither: 4299 (86.2)	1.00	
Prescription medications		
Nervous system medications		
Antidepressants		< 0.001
No: 4390 (88.0)	1.00	
Yes: 596 (12.0)	1.52 (1.28, 1.79), < 0.001	
CVD medications		
Alpha adrenoceptor blockers		0.08
No: 4661 (93.5)	1.00	
Yes: 325 (6.5)	1.24 (0.97, 1.59), 0.08	
Antiarrhythmic medications		0.04
No: 4882 (97.9)	1.00	
Yes: 104 (2.1)	1.51 (1.02, 2.25), 0.04	
Nitrates		0.09
No: 4779 (95.8)	1.00	
Yes: 207 (4.2)	1.29 (0.97, 1.72), 0.09	

A total of 4986 participants were included in the model (97.6% out of 5108)

*HR* hazard ratio, *95% CI* 95% confidence interval, *CVD* cardiovascular disease, *IHB* irregular heartbeat

*Adjusted *p* value from type 3 test

## Discussion

Over a median of 10 years follow-up of 5108 older adults (aged 50–84 years) in New Zealand, one in five (*n* = 1016, 20%) participants were identified as having at least one new non-vertebral fracture, a significant increase from 6% (*n* = 292) observed at a median of 3.3 years follow-up during the clinical trial [28]. We identified several independent risk factors for non-vertebral fractures, including being female, aged 80–84 years, self-reported European/Other ethnicity, low education level (primary school), living with non-family members or living alone, daily alcohol drinking, having a medical history of bone health issues (fall in the last 4 weeks, fracture, osteoporosis and arthritis), and dispensing antidepressants or antiarrhythmic medications. In combination with the high proportion of participants who were living with non-family members or living alone (20.5%), reporting daily alcohol drinking in the last 12 months (21.6%), or taking antidepressants (11.9%), further research into the importance of these factors and public health interventions targeting these potentially modifiable risk factors could have a significant impact (e.g., high population attributable fraction) and be cost-effective for reducing the burden of fractures.

Our findings on socioeconomic factors are largely consistent with literature. The risk of fracture is associated with ethnicity [32] and increases as people age [8, 33], especially for post-menopausal women [6, 33]. The risk of non-vertebral fractures was higher among people with low education (e.g., primary school), which is similar to the results of the CHANCES project, which combined 14 cohorts/studies from Europe and USA [20] and CHARLS, a nationally representative longitudinal survey in China [21]. In addition, similar to the CHANCES project [20], participants living with non-family members or living alone were associated with an increased risk of non-vertebral fractures than those living with family members. The reason may be those people are more likely to experience social isolation, loneliness, and even depressive symptoms [34], which have been associated with an increased risk of fractures [35].

As potential modifiable factors, several lifestyle factors were examined in our cohort. We found a significant dose–response relationship between alcohol drinking frequency and risk of non-vertebral fractures (*p* value for linear trend < 0.001). Daily drinkers exhibited a 43% higher risk of non-vertebral fractures compared to non-drinkers. This dose–response relationship is consistent with results from a meta-analysis of 44 prospective cohort studies [13]. Alcohol and tobacco exposure often coexist [36], and both have been linked to a higher risk of fractures [11, 37]. However, within our cohort, the significant association between tobacco smoking and the risk of non-vertebral fractures disappeared in the final multivariable model. This suggests that the impact of tobacco smoking on non-vertebral fractures may be less important than alcohol drinking in our New Zealand study sample. This could also be attributed to campaigns promoting smoking cessation in New Zealand [38], as evidenced by the observed relatively low prevalence of current smokers (6.3%) and high prevalence of ex-smokers (42.6%) in our sample. Increased physical activity has been linked with a lower risk of fractures, with decreases that varied from 1 to 40% in a review with many studies [39]. However, this association was not confirmed in our study, which may be attributed to the relatively healthy status of the study sample.

Bone health is closely related to the risk of fractures. Falls [18], previous fractures [40], osteoporosis [41] and arthritis [42] are all recognized as risk factors for subsequent fractures. Given the relatively high prevalence of falls and arthritis in our study sample, along with their recognized relationship with fractures, if casualty is confirmed by future studies, population-based interventions that target those risk factors could be cost-effective and eventually reduce the risk of fractures.

Apart from bone health, our study also found a significant relationship between the dispensing of antidepressants and increased risk of non-vertebral fractures, which aligns with the conclusion of meta-analyses on antidepressants and fractures [43]. In addition, antiarrhythmic medications were found to be associated with an increased risk of non-vertebral fractures. Increased risk of fractures has been previously reported for amiodarone [44, 45], likely from increased risk of fall-related injuries and syncope [46], while the findings of digoxin are conflicting [44, 45]. Our finding is consistent with previous studies showing that a history of heart disease contributes to increased fracture risk [21, 47, 48], although a recent meta-analysis showed no association between nitrate use and fractures in observational studies [49]. The effects of specific CVD medications on non-vertebral fractures remain uncertain, and more high-quality, long-term studies are needed to clarify their role in fracture causation.

The strengths of our study include the prospective cohort design, a relatively large sample size (over 5000), and a long follow-up period (10 years). Our study had a broader range of baseline questions and measurements, with few missing data points. The completeness and high quality of the baseline data enabled us to explore a wide range of risk factors within almost all participants. Additionally, the non-vertebral fracture outcome data (adapted from a previous publication from our group [28]) were ascertained by combining two routinely collected databases managed by the MoH and the ACC, ensuring that we could follow up on the occurrence of non-vertebral fractures for all participants. Further, the routine collection of prescriptions by the MoH ensured we had complete data on medications dispensed to the study participants. We employed a systematic domain-based approach for variable selection and conducted sensitivity analyses, all of which enhanced the internal validity of our findings.

There are some limitations in our study. Firstly, the representative of our study sample was compromised by the low response rate (18%) at the recruitment stage among those invited to participate [50], limiting the external validity of our findings. Secondly, BMD, which may explain the increased risk of fracture, was only measured in a subsample of the study (*n* = 413, 8.1%) [51], and was not thoroughly investigated in our study. Although a comprehensive medical history of bone health diagnosis at baseline was collected and analyzed, we were unable to explore factors not collected at our baseline. Furthermore, the majority of the factors of interest were self-reported, which may introduce recall bias.

In summary, among older adults in New Zealand, there has been a high prevalence of non-vertebral fractures. We have identified several risk factors, such as living with non-family members or living alone, reporting daily alcohol drinking in the last 12 months, or taking antidepressants, which are relatively prevalent (> 10%) and potentially modifiable (assuming causality can be established). Further investigation into the causality of these factors and following public health interventions targeting these potentially modifiable risk factors could have a significant impact and be cost-effective for reducing the burden of fractures.

## Supplementary Information

Below is the link to the electronic supplementary material.

Supplementary file1 (DOCX 99 kb)
