# Supplementary material for: Risk factors for non-vertebral fractures in community-dwelling elderly: a 10-year follow-up study in New Zealand
Source: Arch Osteoporos. 2025 Apr 9;20(1):44. doi: 10.1007/s11657-025-01530-7 (PMC11982128; doi:10.1007/s11657-025-01530-7)

## **Supplementary information**

### [Suppl Table](https://dashi.163.com/webmail/read/readhtml.jsp?mid=39:1tbiJwq7MmXAlQfyAwAAsi&userType=ud&font=15&color=3370FF) 1. Types of medications included in the analyses

| **Type of medication** | **Chemical Name** |
| --- | --- |
| **Musculoskeletal medications** |  |
| Non-steroidal anti-inflammatory drugs | Diclofenac sodium, indomethacin, ketoprofen, mefenamic acid, piroxicam, sulindac, tenoxicam, tiaprofenic acid, naproxen, naproxen sodium, ibuprofen or meloxicam. |
| **Nervous system** |  |
| Antiepileptic medication | Lamotrigine, gabapentin, topiramate, carbamazepine, clobazam, clonazepam, diazepam, phenobarbitone sodium, phenytoin sodium, primidone, sodium valproate, phenobarbitone or levetiracetam. |
| Analgesic drugs (non-opioid or opioid analgesics) | Codeine phosphate, dextropropoxyphene with paracetamol, methadone hydrochloride, morphine hydrochloride, morphine sulphate, nefopam hydrochloride, paracetamol with codeine, pethidine hydrochloride, morphine tartrate, dihydrocodeine tartrate, fentanyl or oxycodone hydrochloride. |
| Antidepressants | Amitriptyline, citalopram hydrobromide, clomipramine hydrochloride, dothiepin hydrochloride, doxepin hydrochloride, imipramine hydrochloride, nortriptyline hydrochloride, phenelzine sulphate, tranylcypromine sulphate, trimipramine maleate, fluoxetine hydrochloride, moclobemide, venlafaxine or paroxetine hydrochloride. |
| Antipsychotics | Risperidone, clozapine, olanzapine, quetiapine, chlorpromazine hydrochloride, haloperidol, levomepromazine maleate, trifluoperazine hydrochloride, lithium carbonate or ziprasidone. |
| Anxiolytics | Diazepam, lorazepam, oxazepam, alprazolam or buspirone hydrochloride. |
| Sedatives and hypnotic drugs | Lormetazepam, nitrazepam, temazepam, triazolam, zopiclone or midazolam. |
| **CVD medications** |  |
| Agents affecting the renin-angiotensin system | Losartan potassium, cilazapril with hydrochlorothiazide, candesartan cilexetil, enalapril maleate with hydrochlorothiazide, enalapril maleate, cilazapril, quinapril, lisinopril, perindopril, captopril, or quinapril with hydrochlorothiazide. |
| Alpha adrenoceptor blockers | Prazosin, doxazosin or terazosin drugs. |
| Antiarrhythmic medications | Amiodarone hydrochloride, digoxin, disopyramide phosphate, flecainide acetate, lidocaine hydrochloride or propafenone hydrochloride drugs. |
| Beta adrenoceptor blockers | Atenolol, labetalol, metoprolol succinate, metoprolol tartrate, nadolol, pindolol, propranolol, sotalol, timolol, celiprolol or carvedilol. |
| Calcium channel blockers | Nifedipine, perhexiline maleate, verapamil hydrochloride, felodipine, diltiazem hydrochloride, isradipine or amlodipine. |
| Diuretics | Amiloride hydrochloride, amiloride hydrochloride with furosemide, amiloride hydrochloride with hydrochlorothiazide, bendroflumethiazide [Bendrofluazide], bumetanide, chlortalidone [Chlorthalidone], furosemide [Frusemide], indapamide or spironolactone. |
| Nitrates | Glyceryl trinitrate or isosorbide mononitrate. |
| Lipid lowering agents | Atorvastatin, bezafibrate, cholestyramine, colestipol hydrochloride, gemfibrozil, nicotinic acid, acipimox, simvastatin, pravastatin, ezetimibe or ezetimibe with simvastatin |
| **Other medications** |  |
| **Respiratory Medications** |  |
| Antihistamines | Fexofenadine hydrochloride, chlorpheniramine maleate, dexchlorpheniramine maleate, promethazine hydrochloride, loratadine or cetirizine hydrochloride. |
| Anticholinergic agents | Ipratropium bromide, tiotropium bromide, glycopyrronium or salbutamol with ipratropium bromide. |
| **Alimentary Tract Medications** |  |
| Laxatives | Glycerol, sodium citrate with sodium lauryl sulphoacetate, docusate sodium, poloxamer, lactulose, ispaghula (psyllium) husk, senna, sodium acid phosphate, bisacodyl, magnesium hydroxide, mucilaginous laxatives with stimulants, macrogol 3350 with potassium chloride, sodium bicarbonate and sodium chloride, or docusate sodium with sennosides medications. |

### Suppl Table 2. The diagnosis codes and descriptions of non-vertebral fractures

| **Diagnosis code** | **Descriptions of non-vertebral fractures sites** | **Number** |
| --- | --- | --- |
| **ICD-10 code (from the ACC and MoH)** | |  |
| S02 | skull and face (ACC only) | 9 |
| S22 | ribs and sternum excluding thoracic spine (S220) | 57 |
| S32 | pelvis excluding lumbar spine (S320), sacrum (S321), coccyx (S322) | 19 |
| S42 | shoulder and upper arm | 49 |
| S52 | forearm | 59 |
| S62 | wrist and hand | 46 |
| S72 | femur | 70 |
| S82 | lower leg, including ankle and patella | 53 |
| S92 | foot | 25 |
| T12 | T12 (lower limb, level unspecified). | 0 |
| **Read code (from the ACC)** | |  |
| S01 | skull | 0 |
| S02 | face | 23 |
| S044 | multiple fractures involving skull and facial bones | 1 |
| S10B4 | acetabulum | 1 |
| S12 | rib | 138 |
| S13 | pelvis | 3 |
| S20 | clavicle | 9 |
| S21 | scapula | 3 |
| S22 | humerus | 23 |
| S23 | radius and ulna | 57 |
| S24 | carpal | 51 |
| S25 | metacarpal | 24 |
| S26 | hand phalange | 58 |
| S29 | multiple fractures of clavicle, scapula and humerus (disorder), multiple fractures of forearm; fractures involving multiple regions of both upper limbs | 0 |
| S30 | femoral neck | 17 |
| S31 | other femur | 1 |
| S32 | patella | 6 |
| S33 | tibia and fibula | 14 |
| S34 | ankle | 42 |
| S35 | tarsal and metatarsal | 53 |
| S36 | foot phalange | 85 |
| S3X | lower leg, unspecified part | 1 |
| S4A | shoulder | 2 |
| S4B | elbow | 2 |
| S4C | wrist | 5 |
| S4D | finger or thumb | 3 |
| S4E | hip | 1 |
| S4F | knee | 3 |
| S4G | ankle | 1 |
| S4H | foot | 1 |
| **Unspecified Read code but Read code description clearly indicates non-vertebral fractures (from the ACC)** | | |
| Fracture of skull (S0) | | 0 |
| Fracture of upper limb (S2) | | 1 |
| Fracture of lower limb (S3) | | 0 |

### Suppl Table 3. Lifestyle factors and the hazard ratios of non-vertebral fractures during follow-up

| Lifestyle: n (column%) | Non-vertebral  fractures, n (row %) | Univariate | |  | Multivariable (Model 2) | |  | Multivariable (Model 3) | |
| --- | --- | --- | --- | --- | --- | --- | --- | --- | --- |
|  |  | HR (95% CI), *p* value | *p* value* |  | HR (95% CI), *p* value | *p* value* | | HR (95% CI), *p* value | *p* value* |
| Tobacco smoking |  |  | 0.005 |  |  | 0.003 |  |  | 0.04 |
| Current smoker: 322 (6.3) | 70 (21.7) | 1.27 (0.99, 1.64), 0.06 |  |  | 1.38 (1.07, 1.79), 0.01 |  |  | 1.30 (1.00, 1.69), 0.046 |  |
| Ex-smoker: 2177 (42.6) | 467 (21.5) | 1.22 (1.07, 1.38), 0.002 |  |  | 1.21 (1.06, 1.38), 0.004 |  |  | 1.15 (1.01, 1.31), 0.04 |  |
| Never smoker: 2603 (51.0) | 479 (18.4) | 1.00 |  |  | 1.00 |  |  | 1.00 |  |
| Missing: 6 (0.1) | 0 |  |  |  |  |  |  |  |  |
| Alcohol drinking frequency  in the last 12 months |  |  | <0.001 |  |  | <0.001 |  |  | <0.001 |
| None: 712 (13.9) | 106 (14.9) | 1.00 |  |  | 1.00 |  |  | 1.00 |  |
| < 4 times monthly: 1541 (30.2) | 277 (18.0) | 1.23 (0.98, 1.53), 0.07 |  |  | 1.02 (0.81, 1.28), 0.87 |  |  | 1.02 (0.81, 1.28), 0.87 |  |
| < 7 times weekly: 1745 (34.2) | 345 (19.8) | 1.33 (1.07, 1.66), 0.01 |  |  | 1.13 (0.90, 1.43), 0.29 |  |  | 1.12 (0.89, 1.40), 0.35 |  |
| Daily: 1102 (21.6) | 288 (26.1) | 1.86 (1.49, 2.32), <0.001 |  |  | 1.49 (1.18, 1.89), 0.001 |  |  | 1.44 (1.13, 1.82), 0.003 |  |
| Missing: 8 (0.2) | 0 |  |  |  |  |  |  |  |  |
| Vigorous physical  activity (hours/week) |  |  | 0.30 |  |  | 0.35 |  |  |  |
| None: 2033 (39.8) | 416 (20.5) | 1.00 |  |  | 1.00 |  |  |  |  |
| 0-2: 1235 (24.2) | 234 (18.9) | 0.88 (0.75, 1.03), 0.12 |  |  | 0.92 (0.78, 1.08), 0.29 |  |  |  |  |
| >2: 1636 (32.0) | 335 (20.5) | 0.96 (0.83, 1.10), 0.54 |  |  | 1.04 (0.89, 1.20), 0.65 |  |  |  |  |
| Missing: 204 (4.0) | 31 |  |  |  |  |  |  |  |  |

Notes, n, number of participants; HR, hazard ratio; 95% CI, 95% confidence interval; Model 2: adjusted for all variables in Model 1; Model 3: adjusted all variables in Model 1 and significant variable(s) in Model 2; *, *p* value from Type 3 test.

### Suppl Table 4. Physical health and physical/laboratory measurements factors and the hazard ratios of non-vertebral fractures during follow-up

| Physical health/measurements:  n (column%) | Non-vertebral  fractures, n (row %) | Univariate | |  | Multivariable (Model 2) | |  | Multivariable (Model 3) | |
| --- | --- | --- | --- | --- | --- | --- | --- | --- | --- |
|  |  | HR (95% CI), *p* value | *p* value* |  | HR (95% CI), *p* value | *p* value* | | HR (95% CI), *p* value | *p* value* |
| BMI (Kg/m^2^) |  |  | 0.004 |  |  | 0.29 |  |  |  |
| <18.5: 25 (0.5) | 9 (36.0) | 1.86 (0.96, 3.61), 0.07 |  |  | 1.57 (0.80, 3.05), 0.19 |  |  |  |  |
| 18.5-24.9: 1182 (23.1) | 267 (22.6) | 1.00 |  |  | 1.00 |  |  |  |  |
| 25.0-29.9: 2294 (44.9) | 451 (19.7) | 0.86 (0.74, 1.00), 0.04 |  |  | 0.93 (0.80, 1.09), 0.37 |  |  |  |  |
| ≥30.0: 1583 (31.0) | 285 (18.0) | 0.79 (0.67, 0.93), 0.005 |  |  | 0.90 (0.76, 1.07), 0.24 |  |  |  |  |
| Missing: 24 (0.5) | 4 |  |  |  |  |  |  |  |  |
| Deseasonalized 25(OH)D  concentrations (nmol/L) |  |  | 0.06 |  |  | 0.67 |  |  |  |
| <25.0: 91 (1.8) | 14 (15.4) | 0.70 (0.41, 1.20), 0.19 |  |  | 1.08 (0.62, 1.87), 0.79 |  |  |  |  |
| 25.0- <50.0: 1179 (23.1) | 200 (17.0) | 0.80 (0.68, 0.95), 0.01 |  |  | 0.90 (0.76, 1.08), 0.25 |  |  |  |  |
| 50- <75.0: 2157 (42.2) | 445 (20.6) | 0.95 (0.83, 1.10), 0.51 |  |  | 0.98 (0.85, 1.13), 0.79 |  |  |  |  |
| ≥75.0: 1679 (32.9) | 356 (21.2) | 1.00 |  |  | 1.00 |  |  |  |  |
| Missing: 2 (<0.1) | 1 |  |  |  |  |  |  |  |  |
| Self-reported health status |  |  | 0.21 |  |  | 0.04 |  |  | 0.04 |
| Excellent/Very good: 3857 (75.5) | 763 (19.8) | 1.00 |  |  | 1.00 |  |  | 1.00 |  |
| Good: 1033 (20.2) | 204 (19.7) | 1.04 (0.89, 1.21), 0.65 |  |  | 1.11 (0.95, 1.30), 0.18 |  |  | 1.11 (0.95, 1.30), 0.18 |  |
| Fair/Poor: 212 (4.2) | 49 (23.1) | 1.29 (0.97, 1.73), 0.08 |  |  | 1.40 (1.05, 1.88), 0.02 |  |  | 1.40 (1.05, 1.88), 0.02 |  |
| Missing: 6 (0.1) | 0 |  |  |  |  |  |  |  |  |

Notes, n, number of participants; HR, hazard ratio; 95%CI, 95% confidence interval; 25(OH)D, deseasonalized 25-hydroxyvitaminD; Model 2: adjusted for all variables in Model 1; Model 3: adjusted all variables in Model 1 and significant variable(s) in Model 2; *, *p* value from Type 3 test.

### Suppl Table 5. Medical history factors and the hazard ratios of non-vertebral fractures during follow-up

| Medical history: n (column%) | Non-vertebral  fractures,  n (row %) | Univariate | |  | Multivariable (Model 2) | |  | Multivariable (Model 3) | |
| --- | --- | --- | --- | --- | --- | --- | --- | --- | --- |
|  |  | HR (95% CI), *p* value | *p* value* |  | HR (95% CI), *p* value | *p* value* | | HR (95% CI), *p* value | *p* value* |
| **Bone Health** |  |  |  |  |  |  |  |  |  |
| Fall in the last 4 weeks |  |  | <0.001 |  |  | <0.001 |  |  | <0.001 |
| Yes: 308 (6.0) | 99 (32.1) | 1.81 (1.47, 2.23), <0.001 |  |  | 1.65 (1.34, 2.03), <0.001 |  |  | 1.60 (1.29, 1.98), <0.001 |  |
| No: 4792 (93.8) | 917 (19.1) | 1.00 |  |  | 1.00 |  |  | 1.00 |  |
| Missing: 8 (0.2) | 0 |  |  |  |  |  |  |  |  |
| Previous fracture |  |  | <0.001 |  |  | <0.001 |  |  | <0.001 |
| Yes: 2378 (46.6) | 570 (24.0) | 1.53 (1.35, 1.73), <0.001 |  |  | 1.49 (1.31, 1.68), <0.001 |  |  | 1.44 (1.27, 1.64), <0.001 |  |
| No: 2710 (53.1) | 444 (16.4) | 1.00 |  |  | 1.00 |  |  | 1.00 |  |
| Missing: 20 (0.4) | 2 |  |  |  |  |  |  |  |  |
| Osteoporosis |  |  | <0.001 |  |  | <0.001 |  |  | 0.003 |
| Yes: 71 (1.4) | 29 (40.8) | 2.56 (1.77, 3.71), <0.001 |  |  | 2.04 (1.40, 2.96), <0.001 |  |  | 1.80 (1.23, 2.64), 0.003 |  |
| No: 5016 (98.2) | 983 (19.6) | 1.00 |  |  | 1.00 |  |  | 1.00 |  |
| Missing: 21 (0.4) | 4 |  |  |  |  |  |  |  |  |
| Arthritis |  |  | <0.001 |  |  | <0.001 |  |  | 0.008 |
| Yes: 1755 (34.4) | 419 (23.9) | 1.44 (1.27, 1.63), <0.001 |  |  | 1.32 (1.16, 1.50), <0.001 |  |  | 1.20 (1.05, 1.37), 0.008 |  |
| No: 3315 (64.9) | 587 (17.7) | 1.00 |  |  | 1.00 |  |  | 1.00 |  |
| Missing: 38 (0.7) | 10 |  |  |  |  |  |  |  |  |
| **CVD** |  |  |  |  |  |  |  |  |  |
| Heart attack & Angina |  |  | 0.41 |  |  | 0.46 |  |  |  |
| Heart attack +/- Angina: 367 (7.2) | 67 (18.3) | 1.04 (0.81, 1.33), 0.76 |  |  | 1.07 (0.83, 1.38), 0.60 |  |  |  |  |
| Angina only: 205 (4.0) | 47 (22.9) | 1.22 (0.91, 1.63), 0.19 |  |  | 1.20 (0.89, 1.61), 0.24 |  |  |  |  |
| Neither: 4507 (88.2) | 898 (19.9) | 1.00 |  |  | 1.00 |  |  |  |  |
| Missing: 29 (0.6) | 4 |  |  |  |  |  |  |  |  |
| Heart failure & IHB |  |  | 0.004 |  |  | 0.01 |  |  | 0.04 |
| Heart failure +/- IHB: 84 (1.6) | 19 (22.6) | 1.59 (1.01, 2.50), 0.047 |  |  | 1.58 (1.00, 2.49), 0.051 |  |  | 1.46 (0.92, 2.32), 0.11 |  |
| IHB only: 615 (12.0) | 142 (23.1) | 1.28 (1.08, 1.53), 0.006 |  |  | 1.24 (1.03, 1.48), 0.02 |  |  | 1.21 (1.01, 1.46), 0.04 |  |
| Neither: 4352 (85.2) | 839 (19.3) | 1.00 |  |  | 1.00 |  |  | 1.00 |  |
| Missing: 57 (1.1) | 16 |  |  |  |  |  |  |  |  |
| Stroke & TIA |  |  | 0.001 |  |  | 0.01 |  |  | 0.12 |
| Stroke +/- TIA: 81 (1.6) | 19 (23.5) | 1.33 (0.85, 2.10), 0.22 |  |  | 1.34 (0.85, 2.12), 0.21 |  |  | 1.22 (0.77, 1.93), 0.41 |  |
| TIA only: 163 (3.2) | 48 (29.4) | 1.73 (1.29, 2.31), <0.001 |  |  | 1.53 (1.14, 2.06), 0.005 |  |  | 1.35 (1.00, 1.84), 0.053 |  |
| Neither: 4836 (94.7) | 944 (19.5) | 1.00 |  |  | 1.00 |  |  | 1.00 |  |
| Missing: 28 (0.5) | 5 |  |  |  |  |  |  |  |  |
| Diabetes (with treatment) |  |  | 0.01 |  |  | 0.40 |  |  |  |
| Yes: 576 (11.3) | 87 (15.1) | 0.75 (0.61, 0.94), 0.01 |  |  | 0.91 (0.72, 1.14), 0.40 |  |  |  |  |
| No: 4525 (88.6) | 929 (20.5) | 1.00 |  |  | 1.00 |  |  |  |  |
| Missing: 7 (0.1) | 0 |  |  |  |  |  |  |  |  |
| High blood pressure  (taking pills regularly) |  |  | 0.58 |  |  | 0.96 |  |  |  |
| Yes: 1885 (36.9) | 376 (19.9) | 1.04 (0.91, 1.18), 0.58 |  |  | 1.00 (0.87, 1.14), 0.96 |  |  |  |  |
| No: 3186 (62.4) | 633 (19.9) | 1.00 |  |  | 1.00 |  |  |  |  |
| Missing: 37 (0.7) | 7 |  |  |  |  |  |  |  |  |
| High cholesterol level  (taking pills regularly) |  |  | 0.70 |  |  | 0.99 |  |  |  |
| Yes: 1808 (35.4) | 349 (19.3) | 0.98 (0.86, 1.11), 0.70 |  |  | 1.00 (0.88, 1.14), 0.99 |  |  |  |  |
| No: 3242 (63.5) | 652 (20.1) | 1.00 |  |  | 1.00 |  |  |  |  |
| Missing: 58 (1.1) | 15 |  |  |  |  |  |  |  |  |
| **Other disease** |  |  |  |  |  |  |  |  |  |
| Asthma |  |  | 0.02 |  |  | 0.02 |  |  | 0.16 |
| Yes: 702 (13.7) | 164 (23.4) | 1.23 (1.04, 1.45), 0.02 |  |  | 1.22 (1.03, 1.44), 0.02 |  |  | 1.13 (0.95, 1.35), 0.16 |  |
| No: 4386 (85.9) | 852 (19.4) | 1.00 |  |  | 1.00 |  |  | 1.00 |  |
| Missing: 20 (0.4) | 0 |  |  |  |  |  |  |  |  |
| Emphysema |  |  | 0.31 |  |  | 0.65 |  |  |  |
| Yes: 193 (3.8) | 43 (22.3) | 1.17 (0.86, 1.59), 0.31 |  |  | 1.08 (0.79, 1.46), 0.65 |  |  |  |  |
| No: 4901 (95.9) | 972 (19.8) | 1.00 |  |  | 1.00 |  |  |  |  |
| Missing: 14 (0.3) | 1 |  |  |  |  |  |  |  |  |
| Psoriasis |  |  | 0.43 |  |  | 0.76 |  |  |  |
| Yes: 250 (4.9) | 54 (21.6) | 1.12 (0.85, 1.47), 0.43 |  |  | 1.05 (0.79, 1.38), 0.76 |  |  |  |  |
| No: 4821 (94.4) | 954 (19.8) | 1.00 |  |  | 1.00 |  |  |  |  |
| Missing: 37 (0.7) | 8 |  |  |  |  |  |  |  |  |
| Eczema |  |  | 0.26 |  |  | 0.58 |  |  |  |
| Yes: 600 (11.7) | 130 (21.7) | 1.11 (0.92, 1.34), 0.26 |  |  | 1.05 (0.88, 1.27), 0.58 |  |  |  |  |
| No: 4467 (87.5) | 880 (19.7) | 1.00 |  |  | 1.00 |  |  |  |  |
| Missing: 41 (0.8) | 6 |  |  |  |  |  |  |  |  |
| Cancer |  |  | 0.03 |  |  | 0.77 |  |  |  |
| Yes: 1214 (23.8) | 263 (21.7) | 1.17 (1.02, 1.35), 0.03 |  |  | 1.02 (0.88, 1.18), 0.77 |  |  |  |  |
| No: 3871 (75.8) | 750 (19.4) | 1.00 |  |  | 1.00 |  |  |  |  |
| Missing: 23 (0.5) | 3 |  |  |  |  |  |  |  |  |
| Chronic pain |  |  | <0.001 |  |  | <0.001 |  |  | 0.12 |
| Yes: 884 (17.3) | 219 (24.8) | 1.37 (1.18, 1.59), <0.001 |  |  | 1.31 (1.13, 1.53), <0.001 |  |  | 1.14 (0.97, 1.33), 0.12 |  |
| No: 4217 (82.6) | 797 (18.9) | 1.00 |  |  | 1.00 |  |  | 1.00 |  |
| Missing: 7 (0.1) | 0 |  |  |  |  |  |  |  |  |
| Depression (lasted for  more than 6 months) |  |  | <0.001 |  |  | 0.02 |  |  | 0.16 |
| Yes: 536 (10.5) | 137 (25.6) | 1.38 (1.16, 1.66), <0.001 |  |  | 1.24 (1.03, 1.49), 0.02 |  |  | 1.15 (0.95, 1.38), 0.16 |  |
| No: 4560 (89.3) | 878 (19.3) | 1.00 |  |  | 1.00 |  |  | 1.00 |  |
| Missing: 12 (0.2) | 1 |  |  |  |  |  |  |  |  |

Notes, n, number of participants; HR, hazard ratio; 95% CI, 95% confidence interval; CVD, cardiovascular disease; IHB, irregular heart beat; TIA, transient ischaemic attack; Model 2: adjusted for all variables in Model 1; Model 3: adjusted all variables in Model 1 and significant variable(s) in Model 2; *p* value from Type 3 test.

### Suppl Table 6. Medication factors and the hazard ratios of non-vertebral fractures during follow-up

| Prescription Medications:  n (column%) | Non-vertebral fractures,  n (row %) | Univariate | |  | Model 2 | |  | Model 3 | |
| --- | --- | --- | --- | --- | --- | --- | --- | --- | --- |
|  |  | HR (95% CI), *p* value | *p* value* |  | HR (95% CI), *p* value | *p* value* | | HR (95% CI), *p* value | *p* value* |
| **Musculoskeletal medications** |  |  |  |  |  |  |  |  |  |
| Non-steroidal  anti-inflammatory drugs |  |  | 0.23 |  |  | 0.06 |  |  | 0.36 |
| No: 3793 (74.3) | 739 (19.5) | 1.00 |  |  | 1.00 |  |  | 1.00 |  |
| Yes: 1315 (25.7) | 277 (21.1) | 1.09 (0.95, 1.25), 0.23 |  |  | 1.14 (0.99, 1.21), 0.06 |  |  | 1.07 (0.93, 1.23), 0.36 |  |
| **Nervous system medications** |  |  |  |  |  |  |  |  |  |
| Antiepileptic medication |  |  | <0.001 |  |  | 0.003 |  |  | 0.11 |
| No: 4906 (96.0) | 958 (19.5) | 1.00 |  |  | 1.00 |  |  | 1.00 |  |
| Yes: 202 (4.0) | 58 (28.7) | 1.61 (1.24, 2.10), <0.001 |  |  | 1.51 (1.15, 1.97), 0.003 |  |  | 1.28 (0.95, 1.72), 0.11 |  |
| Analgesic drugs (non-opioid  or opioid analgesics) |  |  | 0.004 |  |  | 0.002 |  |  | 0.16 |
| No: 4448 (87.1) | 861 (19.4) | 1.00 |  |  | 1.00 |  |  | 1.00 |  |
| Yes: 660 (12.9) | 155 (23.5) | 1.29 (1.08, 1.53), 0.004 |  |  | 1.31 (1.10, 1.55), 0.002 |  |  | 1.14 (0.95, 1.37), 0.16 |  |
| Antidepressants |  |  | <0.001 |  |  | <0.001 |  |  | <0.001 |
| No: 4498 (88.1) | 830 (18.5) | 1.00 |  |  | 1.00 |  |  | 1.00 |  |
| Yes: 610 (11.9) | 186 (30.5) | 1.78 (1.52, 2.09), <0.001 |  |  | 1.59 (1.35, 1.87), <0.001 |  |  | 1.45 (1.22, 1.72), <0.001 |  |
| Antipsychotics |  |  | 0.19 |  |  | 0.36 |  |  |  |
| No: 5050 (98.9) | 1001 (19.8) | 1.00 |  |  | 1.00 |  |  |  |  |
| Yes: 58 (1.1) | 15 (25.9) | 1.41 (0.84, 2.34), 0.19 |  |  | 1.27 (0.76, 2.13), 0.36 |  |  |  |  |
| Anxiolytics |  |  | 0.01 |  |  | 0.10 |  |  | 1.00 |
| No: 4962 (97.1) | 975 (19.6) | 1.00 |  |  | 1.00 |  |  | 1.00 |  |
| Yes: 146 (2.9) | 41 (28.1) | 1.49 (1.09, 2.03), 0.01 |  |  | 1.31 (0.95, 1.79), 0.10 |  |  | 1.00 (0.71, 1.41), 1.00 |  |
| Sedatives and hypnotic drugs |  |  | <0.001 |  |  | 0.05 |  |  | 0.45 |
| No: 4537 (88.8) | 869 (19.2) | 1.00 |  |  | 1.00 |  |  | 1.00 |  |
| Yes: 571 (11.2) | 147 (25.7) | 1.39 (1.17, 1.65), <0.001 |  |  | 1.19 (1.00, 1.42), 0.05 |  |  | 1.07 (0.90, 1.29), 0.45 |  |
| **CVD medications** |  |  |  |  |  |  |  |  |  |
| Agents affecting the  renin-angiotensin system |  |  | 0.92 |  |  | 0.55 |  |  |  |
| No: 3350 (65.6) | 674 (20.1) | 1.00 |  |  | 1.00 |  |  |  |  |
| Yes: 1758 (34.4) | 342 (19.5) | 1.01 (0.88, 1.15), 0.92 |  |  | 1.04 (0.91, 1.19), 0.55 |  |  |  |  |
| Alpha adrenoceptor blockers |  |  | 0.03 |  |  | 0.03 |  |  | 0.06 |
| No: 4775 (93.5) | 939 (19.7) | 1.00 |  |  | 1.00 |  |  | 1.00 |  |
| Yes: 333 (6.5) | 77 (23.1) | 1.29 (1.02, 1.63), 0.03 |  |  | 1.31 (1.03, 1.66), 0.03 |  |  | 1.26 (0.99, 1.60), 0.06 |  |
| Antiarrhythmic medications |  |  | 0.008 |  |  | 0.01 |  |  | 0.03 |
| No: 5001 (97.9) | 987 (19.7) | 1.00 |  |  | 1.00 |  |  | 1.00 |  |
| Yes: 107 (2.1) | 29 (27.1) | 1.65 (1.14, 2.38), 0.008 |  |  | 1.60 (1.10, 2.32), 0.01 |  |  | 1.52 (1.05, 2.21), 0.03 |  |
| Beta adrenoceptor blockers |  |  | 0.28 |  |  | 0.51 |  |  |  |
| No: 4079 (79.9) | 807 (19.8) | 1.00 |  |  | 1.00 |  |  |  |  |
| Yes: 1029 (20.1) | 209 (20.3) | 1.09 (0.93, 1.27), 0.28 |  |  | 1.05 (0.90, 1.23), 0.51 |  |  |  |  |
| Calcium channel blockers |  |  | 0.16 |  |  | 0.23 |  |  |  |
| No: 4244 (83.1) | 837 (19.7) | 1.00 |  |  | 1.00 |  |  |  |  |
| Yes: 864 (16.9) | 179 (20.7) | 1.12 (0.96, 1.32), 0.16 |  |  | 1.11 (0.94, 1.31), 0.23 |  |  |  |  |
| Diuretics |  |  | 0.02 |  |  | 0.53 |  |  |  |
| No: 4454 (87.2) | 872 (19.6) | 1.00 |  |  | 1.00 |  |  |  |  |
| Yes: 654 (12.8) | 144 (22.0) | 1.24 (1.04, 1.48), 0.02 |  |  | 1.06 (0.88, 1.27), 0.53 |  |  |  |  |
| Nitrates |  |  | 0.001 |  |  | 0.005 |  |  | 0.02 |
| No: 4891 (95.8) | 959 (19.6) | 1.00 |  |  | 1.00 |  |  | 1.00 |  |
| Yes: 217 (4.2) | 57 (26.3) | 1.55 (1.19, 2.02), 0.001 |  |  | 1.47 (1.12, 1.93), 0.005 |  |  | 1.39 (1.06, 1.83), 0.02 |  |
| Lipid lowering agents |  |  | 0.72 |  |  | 0.39 |  |  |  |
| No: 2835 (55.5) | 566 (20.0) | 1.00 |  |  | 1.00 |  |  |  |  |
| Yes: 2273 (44.5) | 450 (19.8) | 1.02 (0.90, 1.16), 0.72 |  |  | 1.06 (0.93, 1.20), 0.39 |  |  |  |  |
| **Other medications** |  |  |  |  |  |  |  |  |  |
| **Respiratory medications** |  |  |  |  |  |  |  |  |  |
| Agents affecting the  renin-angiotensin system |  |  | 0.92 |  |  | 0.48 |  |  |  |
| No: 3350 (65.6) | 674 (20.1) | 1.00 |  |  | 1.00 |  |  |  |  |
| Yes: 1758 (34.4) | 342 (19.5) | 1.01 (0.88, 1.15), 0.92 |  |  | 1.07 (0.89, 1.29), 0.48 |  |  |  |  |
| Alpha adrenoceptor blockers |  |  | 0.03 |  |  | 0.02 |  |  | 0.11 |
| No: 4775 (93.5) | 939 (19.7) | 1.00 |  |  | 1.00 |  |  | 1.00 |  |
| Yes: 333 (6.5) | 77 (23.1) | 1.29 (1.02, 1.63), 0.03 |  |  | 1.46 (1.08, 1.99), 0.02 |  |  | 1.29 (0.95, 1.76), 0.11 |  |
| **Alimentary tract medications** |  |  |  |  |  |  |  |  |  |
| Laxatives |  |  | <0.001 |  |  | 0.002 |  |  | 0.14 |
| No: 4669 (91.4) | 902 (19.3) | 1.00 |  |  | 1.00 |  |  | 1.00 |  |
| Yes: 439 (8.6) | 114 (26.0) | 1.47 (1.21, 1.79), <0.001 |  |  | 1.38 (1.13, 1.68), 0.002 |  |  | 1.17 (0.95, 1.44), 0.14 |  |

Notes, n, number of participants; HR, hazard ratio; 95%CI, 95% confidence interval; CVD, cardiovascular disease; Model 2: adjusted for all variables in Model 1; Model 3: adjusted all variables in Model 1 and significant variable(s) in Model 2; *p* value from Type 3 test.

### Suppl Table 7. The sensitivity analysis of multivariable Cox model and adjusted hazard ratios of non-vertebral fractures during follow-up

| Characteristic | Sensitivity analysis * | |
| --- | --- | --- |
|  | HR (95% CI), *p* value | *p* value*#* |
| **Sociodemographic** |  |  |
| Sex |  | <0.001 |
| Female | 1.47 (1.29, 1.68), <0.001 |  |
| Male | 1.00 |  |
| Age (years) |  | <0.001 |
| 50-59 | 1.00 |  |
| 60-69 | 0.87 (0.73, 1.04), 0.13 |  |
| 70-79 | 0.94 (0.77, 1.14), 0.52 |  |
| 80-84 | 1.50 (1.15, 1.96), 0.003 |  |
| Ethnic group |  | <0.001 |
| European/Other | 1.00 |  |
| Māori | 0.61 (0.43, 0.87), 0.006 |  |
| Pacific | 0.45 (0.30, 0.68), <0.001 |  |
| South Asian | 0.50 (0.32, 0.80), 0.003 |  |
| Highest education level |  | 0.04 |
| Primary school | 1.71 (1.13, 2.61), 0.01 |  |
| Secondary school | 1.04 (0.91, 1.19), 0.54 |  |
| Tertiary (eg. university) | 1.00 |  |
| Living situation |  | 0.001 |
| Family members | 1.00 |  |
| Non-family members | 1.51 (1.04, 2.20), 0.03 |  |
| Alone | 1.31 (1.12, 1.53), 0.001 |  |
| **Lifestyle** |  |  |
| Alcohol drinking frequency in the last 12 months |  | <0.001 |
| None | 1.00 |  |
| < 4 times monthly | 1.06 (0.84, 1.34), 0.64 |  |
| < 7 times weekly | 1.19 (0.94, 1.51), 0.14 |  |
| Daily | 1.54 (1.21, 1.95), <0.001 |  |
| **Physical health** |  |  |
| **Medical History** |  |  |
| **Bone Health** |  |  |
| Fall in the last 4 weeks |  | <0.001 |
| Yes | 1.61 (1.30, 1.98), <0.001 |  |
| No | 1.00 |  |
| Previous fracture |  | <0.001 |
| Yes | 1.44 (1.27, 1.64), <0.001 |  |
| No | 1.00 |  |
| Osteoporosis |  | 0.001 |
| Yes | 1.95 (1.33, 2.86), 0.001 |  |
| No | 1.00 |  |
| Arthritis |  | 0.004 |
| Yes | 1.21 (1.06, 1.38), <0.004 |  |
| No | 1.00 |  |
| **Prescription Medications** |  |  |
| **Nervous system medications** |  |  |
| Antidepressants |  | <0.001 |
| No | 1.00 |  |
| Yes | 1.55 (1.31, 1.83), <0.001 |  |
| **CVD medications** |  |  |
| Antiarrhythmic medications |  | 0.004 |
| No | 1.00 |  |
| Yes | 1.72 (1.18, 2.49), 0.004 |  |
| Nitrates |  | 0.04 |
| No | 1.00 |  |
| Yes | 1.35 (1.02, 1.79), 0.04 |  |

Notes, HR, hazard ratio; 95%CI, 95% confidence interval; CVD, cardiovascular disease; A total of 4985 participants were included in the model (97.6% out of 5108); *, the stepwise method was used for selecting risk factors; #, p value from Type 3 test.

### Suppl Figure 1. The dose-response relationship between the alcohol drinking frequency and the adjusted risk of non-vertebral fractures.


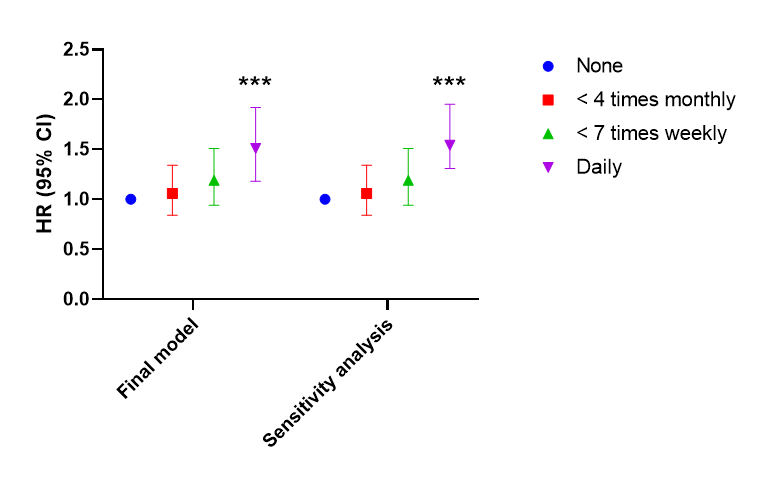

Supplement: Supplementary file 1 — Supplementary file1 (DOCX 99 kb) [file 11657_2025_1530_MOESM1_ESM.docx]
